# Application of Deep Learning in the Identification of Cerebral Hemodynamics Data Obtained from Functional Near-Infrared Spectroscopy: A Preliminary Study of Pre- and Post-Tooth Clenching Assessment

**DOI:** 10.3390/jcm9113475

**Published:** 2020-10-28

**Authors:** Shinya Takagi, Shigemitsu Sakuma, Ichizo Morita, Eri Sugimoto, Yoshihiro Yamaguchi, Naoya Higuchi, Kyoko Inamoto, Yoshiko Ariji, Eiichiro Ariji, Hiroshi Murakami

**Affiliations:** 1 Department of Fixed Prosthodontics, School of Dentistry, Aichi Gakuin University, Nagoya 464-8651, Japan; yy0201@dpc.agu.ac.jp; 2 Japanese Red Cross Toyota College of Nursing, Toyota 471-8565, Japan; i-morita@rctoyota.ac.jp; 3 Department of Pediatric Dentistry, School of Dentistry, Aichi Gakuin University, Nagoya 464-8651, Japan; motimoti1026@gmail.com; 4 Department of Endodontics, School of Dentistry, Aichi Gakuin University, Nagoya 464-8651, Japan; kinchan@dpc.aichi-gakuin.ac.jp (N.H.); kyon@dpc.agu.ac.jp (K.I.); 5 Department of Oral and Maxillofacial Radiology, School of Dentistry, Aichi Gakuin University, Nagoya 464-8651, Japan; yoshiko@dpc.agu.ac.jp (Y.A.); ariji@dpc.agu.ac.jp (E.A.); 6 Department of Gerodontology and Home Care Dentistry, School of Dentistry, Aichi Gakuin University, Nagoya 464-8651, Japan; hiroshi@dpc.agu.ac.jp

**Keywords:** deep learning, deoxy-hemoglobin, functional near-infrared spectroscopy, oxy-hemoglobin

## Abstract

In fields using functional near-infrared spectroscopy (fNIRS), there is a need for an easy-to-understand method that allows visual presentation and rapid analysis of data and test results. This preliminary study examined whether deep learning (DL) could be applied to the analysis of fNIRS-derived brain activity data. To create a visual presentation of the data, an imaging program was developed for the analysis of hemoglobin (Hb) data from the prefrontal cortex in healthy volunteers, obtained by fNIRS before and after tooth clenching. Three types of imaging data were prepared: oxygenated hemoglobin (oxy-Hb) data, deoxygenated hemoglobin (deoxy-Hb) data, and mixed data (using both oxy-Hb and deoxy-Hb data). To differentiate between rest and tooth clenching, a cross-validation test using the image data for DL and a convolutional neural network was performed. The network identification rate using Hb imaging data was relatively high (80‒90%). These results demonstrated that a method using DL for the assessment of fNIRS imaging data may provide a useful analysis system.

## 1. Introduction

With the aging of society, degradation of the quality of life due to a decline in cognitive function (i.e., deterioration of memory and judgment ability) is becoming a severe social problem. It has been reported that efforts in daily activities such as exercise (e.g., exercise to improve oral function) may prevent a decline in cognitive function [1,2,3]. We hypothesized that functional movement of the stomatognathic system may help to maintain and improve cognitive function, and we have therefore studied cerebral hemodynamics of the prefrontal cortex during mastication and tooth clenching, using functional near-infrared spectroscopy (fNIRS). In our previous studies [4,5], we reported increased brain activation of the dorsolateral prefrontal cortex (DLPFC) during gum chewing and tooth clenching in healthy individuals.

Brain function measurement techniques include magnetic resonance imaging (MRI), positron emission tomography, and magnetoencephalography. However, these methods have the disadvantages of utilizing fixed devices that require head fixation to reduce movement artifacts during measurements and/or involve the risk of radiation exposure. On the other hand, fNIRS is noninvasive and can be used repeatedly in a wide range of age groups, from infants to the elderly. In addition, as fNIRS does not require head fixation, it provides a high degree of freedom in terms of the posture of the subject during measurement, with a choice of movements that can be studied and a relatively high temporal resolution. In addition, the device itself is compact and portable, making fNIRS a useful brain function-imaging technique [6,7]. fNIRS is being considered for various applications such as diagnosis aid for mental illness [8], evaluation of infant brain function development [9], monitoring of cerebral circulation during general anesthesia [10], and determination of the effect on brain function during rehabilitation [11].

However, fNIRS has some disadvantages; particularly the amount of time required for the complicated data processing necessary to assess changes in brain activation, which has hampered its use for on-site assessment. To maximize the advantage of near real-time data acquisition by fNIRS, it is important to develop a system that allows quick assessment of the effects of exercise and therapy in treatment and laboratory facilities. The data provided by fNIRS are simply a list of numbers; this data format makes it difficult to perform visual identification of active sites and is not appropriate for providing explanations to subjects. Thus, a new assessment system that allows a more user-friendly visual presentation of test data is desirable.

Deep learning (DL) is a learning method that uses an artificial neural network that mimics the neural network of the brain with an automatic feature extraction layer added [12,13]. Of these, convolutional neural networks (CNNs) are often used in the field of image recognition. A typical CNN consists of an input layer, a convolutional layer, a pooling layer, and a fully connected layer. By inputting image data, features can be detected automatically, and images that match the features can be identified [14].

Automatic organ differentiation techniques using CNN on images obtained by computed tomography and MRI have recently been established in the medical field [15,16]. It has been reported that the application of these techniques may facilitate the diagnostic imaging of brain tumors [17,18] and cervical lymph node metastases [19] of brain tumors and oral cancers.

Consequently, we have developed a system that allows rapid assessment and visual presentation of fNIRS data to discern the presence or absence of brain activation based on DL.

By clinically operating this system in the future, it will be possible to immediately determine the effect of treatment, such as rehabilitation, and it will be easy to visually explain it to the patient. In addition, the fact that the measurement result can be judged immediately will be useful in cases such as those when remeasurement is necessary due to a measurement error.

This preliminary study examined the feasibility of using this DL method for differentiating between rest and tooth clenching on fNIRS data converted into hemoglobin (Hb) data.

## 2. Materials and Methods

### 2.1. Subjects

Fifteen healthy volunteers with normal stomatognathic function (11 males and 4 females with a mean age of 27.6 ± 4.4 years), who provided informed consent after being informed of the purpose of the study, were enrolled.

This study was performed with the approval of the Ethics Committee of the School of Dentistry, Aichi-Gakuin University (approval number: 571).

### 2.2. Exercise Task

Gibbs et al. [20] reported that the average bite force exerted at the intercuspal position during tooth clenching is approximately 40% of the maximum bite force. In this study, 40% maximum voluntary contraction (MVC) clenching motion, which can be sustained for a set period of time and is exerted during masticatory motion, was adopted as a task motion, assuming future application for rehabilitation. A myoelectric biofeedback unit (MA-2000W: Osaka Electronic Equipment Ltd, Hiroshima, Japan) was used to control the bite strength. In addition, a surface electrode was attached to the central part of the left masseter muscle for regulation, and maximum clenching was performed and recorded. Clenching was performed while the display part of the myoelectric feedback unit was checked so that the MVC was 40%. The preparation required for the regulation of the clenching strength was performed before starting the measurement.

**Figure 1:**
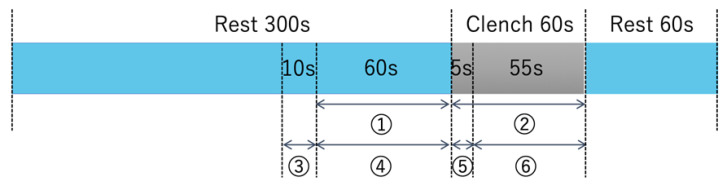
Time course of measurement. Rest 300 s: 300 s at rest, with the subject in sitting position, immediately before “Clench.” Clench: At 40% MVC, while in sitting position, the subject performs a 60 s tooth clenching task at 40% MVC. Rest 60s: 60 s at rest, with the subject in sitting position, immediately following “Clench.” Brain activity was evaluated using hemoglobin data, as follows: (**1**) Rest period: rest (60 s) immediately before the tooth clenching task; (**2**) tooth clenching period: tooth clenching task performed at 40% MVC for 60 s. Brain activity was also evaluated using a deep learning method, as follows: (**3**) Baseline period: 10 s immediately before the rest period (**4**), was used to determine the baseline; (**4**) rest period: a rest of 60 s immediately prior to performing the tooth clenching task; (**5**) exclusion period: 5 s after the start of the tooth clenching task was excluded; (**6**) tooth clenching: the remaining 55 s of the tooth clenching task, performed at 40% MVC, after excluding the first 5 s (exclusion period).

The time course for measurement was as follows: 5 min of rest followed by a 1-min task of tooth clenching at 40% MVC and a 1-min final rest (Figure 1). For instructions on clenching exercise and rest, “Clench” and “Rest” were displayed on the monitor, and the instructions were given visually.

### 2.3. Measurement of Brain Function Using Functional Near-Infrared Spectroscopy (fNIRS) 

This study used a multichannel fNIRS system (ETG-4000, Hitachi Medical Corporation, Tokyo, Japan). Because of the grid pattern arrangement of the near-infrared light irradiation and the light-receiving units, if light is irradiated from the irradiation unit at the same time, all the light-receiving units are simultaneously received, and it becomes difficult to identify the part. Therefore, the modulation/lock-in method was adopted, and two wavelengths (695 and 830 nm) were used for each irradiation position to both extract a specific frequency and simultaneously measure all signals [21]. fNIRS uses two near-infrared wavelengths to measure changes in the concentration of cerebral oxygenated hemoglobin (oxy-Hb) and deoxygenated hemoglobin (deoxy-Hb) [22]. In addition, fNIRS indirectly assesses brain activity based on the neurovascular coupling phenomenon [23], in which an increase in local brain activity leads to an increase in oxygen and glucose consumption, resulting in an increase in cerebral blood flow. Furthermore, because heart rate, respiratory rate, the Mayer wave, and other factors might cause artifacts that affect the analysis of fNIRS signals [24], a 0.2-Hz low-pass filter was applied during measurements. The sampling time was set to 10 Hz. The acquired Hb data were set as common data in “2.4. Evaluation of brain activity using Hb data” and “2.5. Evaluation of brain activity using DL.”

Regarding the relationship between the channel and the anatomical parts of the brain, a virtual registration method [25,26] was used to label sites based on the International 10‒20 system for EEG electrode placement. In the present study, to measure and examine changes in tooth clenching-related brain activity in the prefrontal cortex, measurements were performed using a 22-channel probe corresponding to the area. The near-infrared light was transmitted by an optical fiber to the emission probe positioned on the surface of the scalp, and the reflection of the light was received by the detector probe placed 3.0 cm away from the emission probe. In addition, we used an optical fiber array with 3 rows and 5 columns. There were 7 emission probes and 8 detector probes. Channels (Ch) 2, 3, 7, 12, 16, and 21 correspond to the superior frontal gyrus (SFG), while Ch 1, 4, 5, 6, 8, 9, 10, 11, 13, 15, 17, 20, and 22 correspond to the middle frontal gyrus (MFG). Ch 14, 18, and 19 correspond to the inferior frontal gyrus (IFG).

### 2.4. Evaluation of Brain Activity Using Hemoglobin (Hb) Data

The purpose of this study was to investigate whether the clenching and resting data acquired by fNIRS can be identified by deep learning. Therefore, we first evaluated the presence or absence of brain activity based on the conventional Hb data and examined whether the data acquired this time would produce a data set worthy of DL.

To evaluate brain activity using Hb data, Hb data obtained during rest, immediately before tooth clenching (1 min) (Figure 1) (1) and during tooth clenching (1 min) (Figure 1) (2) were used. To evaluate an increase or decrease in Hb data during tooth clenching, mean values of oxy-Hb and deoxy-Hb data for rest (1 min) and tooth clenching (1 min) were calculated (*n* = 600, 10 Hz). Then, changes for each channel were calculated by subtracting the value for the duration of the rest period from the value for the duration of tooth clenching. The difference in brain activity between tooth clenching and rest was evaluated for each channel using paired *t*-tests, followed by a Bonferroni post-hoc test. For statistical analysis of fNIRS data, paired *t*-tests and ANOVA are often used. ANOVA is often used for the comparison of three or more counties. However, since this study is a comparison of data from two counties, and the paired *t*-test has been adopted in many papers dealing with such a scenario, the results obtained are easy to compare with other reports [27,28,29]. Therefore, in this study, a paired *t*-test was adopted as a statistical method. All statistical analyses were performed using IBM SPSS Statistics version 26.0 for Windows (IBM Corp., Armonk, NY, USA).

### 2.5. Evaluation of Brain Activity Using Deep Learning (DL)

Brain activity during tooth clenching was evaluated using DL by recording data over 55 seconds starting from 5 seconds after the beginning of tooth clenching; data for the duration of the rest period (1 min) immediately before tooth clenching were used as rest period data. The first 5 s were excluded because changes in cerebral hemodynamics lagged somewhat behind the start of tooth clenching [30].

**Figure 2:**
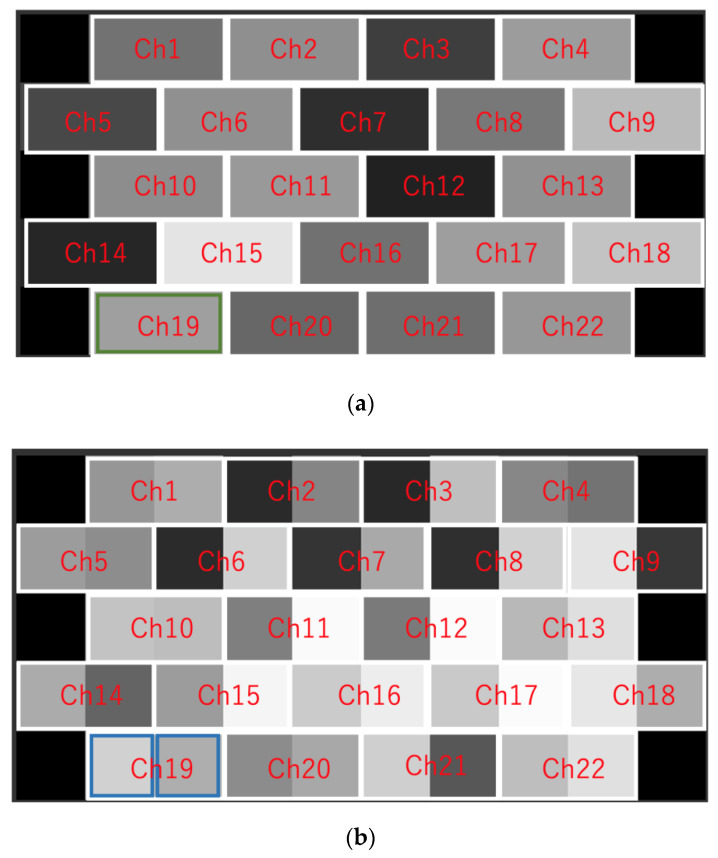
(**a**) A representative oxy image. (**b**) A representative OD image. An image of 5 × 10 pixels was created, and 1 × 2 pixels were assigned to each channel (Ch). The images were converted into gray scale images by 256 gradations, with a maximum value of 255 (white) and a minimum value of 0 (black). As exemplified in (**a**), in the gray scale image of oxy-Hb data or deoxy-Hb data, 1 × 2 pixels represented one cell (green frame, Ch 19). As shown in (**b**), the 1 × 2 pixel is divided in half (blue frame, Ch 19), showing the grayscale image of oxy-Hb data on the left side and deoxy-Hb data on the right side.

**Figure 3:**
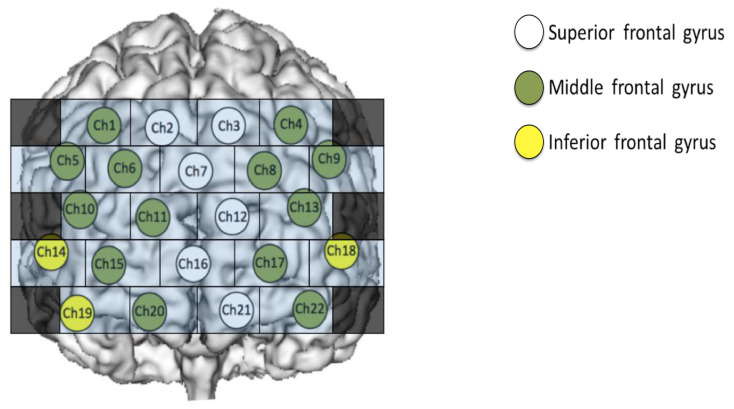
Correspondence between brain regions and images. It is assumed that the Hb image data and the channel (Ch) shown in Figure 2 correspond.

When creating an image from Hb data, we built a program using the functions of Visual Basic for Applications of Microsoft Excel for office365 (Microsoft, Redmond, WA, USA) and created it automatically. Imaging was performed at a sampling rate of 10 Hz, based on the time course shown in Figure 1, as follows: (1) As the mean value of the baseline data, the mean values of the oxy-Hb and deoxy-Hb data obtained during the baseline period (Figure 1) (3) were calculated for each channel for each subject. (2) For every 10 Hz, the oxy-Hb and deoxy-Hb data obtained for the duration of the rest period (Figure 1) (4) and during tooth clenching (Figure 1) (6), respectively, were used to calculate changes from the baseline. The standard deviations (SDs) of the rest period and tooth clenching data were also calculated. In fNIRS measurement, artifacts (e.g., effects of optical fiber contact failure caused by body movement, and changes in muscle blood flow related to the muscle activity) may produce changes that are not related to brain activity [31,32]. Therefore, to remove artifacts as much as possible, we considered values exceeding 2 S.D. in the obtained Hb data as artifacts and treated them as missing values. In the method using DL, the image data acquired at 10Hz was used for DL one by one. We considered the possibility of incorrect learning if the artifacts generated by body movements and muscle blood flow were DL, as indeed they were. Therefore, the values outside the 2S.D. range were regarded as artifacts and treated as missing values. In DL by recurrent neural networks, data were treated in time series, but in DL by CNN, the learning data were shuffled to improve learning accuracy. Therefore, we considered that the learning effect is not affected even if there is no continuity of the data obtained by fNIRS. For this reason, in this study, we decided to treat the data regarded as artifacts as missing values. (3) The rest-period and tooth clenching data calculated in (2) for each subject were used to create grayscale images by converting them into 256 gradations with a maximum value of 255 (white) and a minimum value of 0 (black). For imaging, oxy-Hb data only, deoxy-Hb data only, and mixed data (created using both oxy-Hb and deoxy-Hb data; OD data) were used. To create the oxy image and the deoxy image, oxy-Hb data or deoxy-Hb data were assigned 1 × 2 pixels for each Ch, and this was defined as 1 square, and an image of 5 × 10 pixels was displayed in grayscale (Figure 2a). In addition, considering the possibility of improving the identification rate by simultaneously downloading oxy-Hb data and deoxy-Hb data, 1 × 2 pixels were divided into two: oxy-Hb data on the left side and deoxy-Hb data on the right side. An OD image was created by displaying it and having information on both oxy-Hb and deoxy-Hb in one image (Figure 2b). Hb image data with missing values were excluded from the analysis. The array of images created in this way was compared with channels corresponding to brain regions, as shown in Figure 3. The total number of oxy images created with this procedure was 13,477 (7038 images obtained during the rest period and 6439 images obtained during tooth clenching). The total number of deoxy images was 11,268 (5967 images obtained during the rest period and 5301 images obtained during tooth clenching). The total number of OD images was 9869 (5148 images obtained during the rest period and 4721 obtained during tooth clenching). It has been pointed out that neural network learning with imbalanced training data may affect learning performance [33]. Therefore, subjects were randomly counterbalanced so that the number of images for the duration of the rest period or tooth clenching was equal between the two groups with a higher and lower number of images. Before DL was performed, the image was resized to 256 × 256 pixels using the squash technique in DIGITS 5 (NVIDIA Corporation, Santa Clara, CA, USA).

For DL, we used the Ubuntu 16.04 LTS; Processor, Intel Core i7-6950X CPU (Intel Corporation, Santa Clara, CA, USA) operating system, and the GTX 1080 Ti 11GB (NVIDIA Corporation, Santa Clara, CA, USA) graphics board. To identify the images, we used DL with a convolutional neural network.

**Figure 4:**
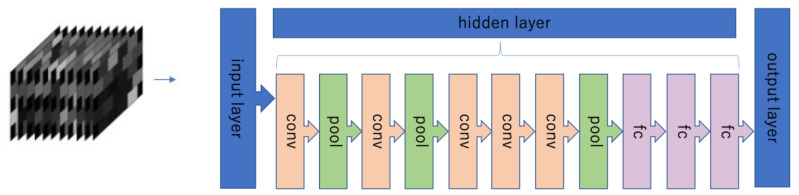
Structure of neural network (AlexNet). The input layer is that of the created image (input data were imaged from the data obtained at a sampling time of 10 Hz by fNIRS); the convolutional layers (conv) are layers that extract image features through a kernel; the max pooling layer (pool) is a layer that emphasizes features by reducing image size; the fully connected layer (fc) is a layer that merges the output from the pooling layer and sends it to the output layer.

AlexNet was used for the network configuration. AlexNet, consisting of five convolutional layers, three maximum pooling layers, and three fully connected layers, has been reported to have high image learning ability (Figure 4) [34].

**Figure 5:**
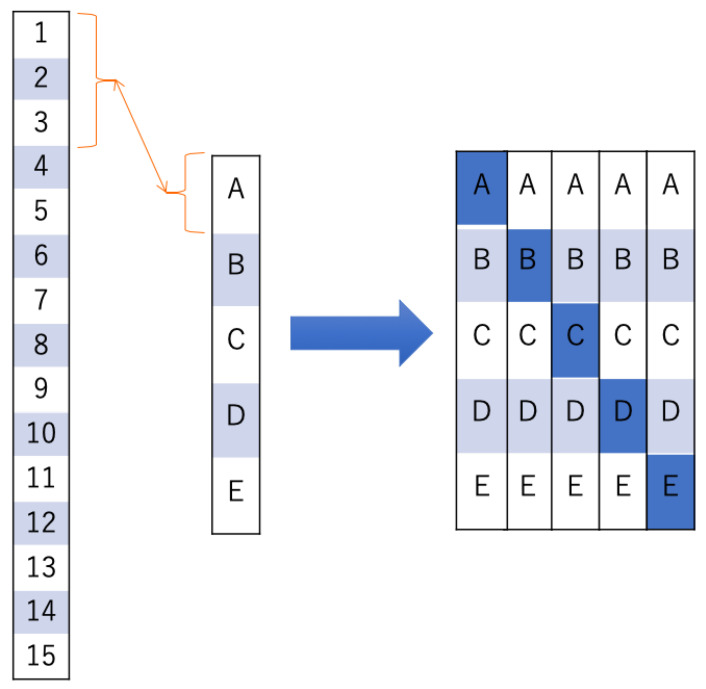
Procedure of the five-fold cross-validation. First, for each of five groups (group A to E), three subjects were randomly selected from among 15 subjects. Using the five-fold cross-validation approach, these five groups were further divided into a training group (consisting of four groups) for deep learning (DL) and a validation group (shown in dark blue above). The process was repeated so that each of the groups was used in DL.

For oxy, deoxy, and OD images, this study used five groups with three subjects randomly selected from among the 15 subjects in each group. A five-fold cross-validation was performed in the five subject groups (i.e., four groups were used as training data and the remaining group was used as test data) (Figure 5). Learning was performed until the learning loss was sufficiently low after 200 epochs. Based on Table 1, the results of identification were calculated as follows: Accuracy = (TN + TP)/(TN + TP + FN + FP), Recall = TP/(TP + FN), Specificity = TN/(FP + TN), Precision = TP/(FP + TP), F-value = (2 × Recall × Precision)/(Recall + Precision). In addition, the mean and SD were calculated for each of the five groups.

Furthermore, a receiver operating characteristic (ROC) curve was created using the correct and incorrect data for oxy, deoxy, and OD images, and analysis was performed using the area under the ROC curve (AUC). The accuracy of identification between the neural networks after learning using oxy, deoxy, and OD images was also compared using the Ryan method (R version 3.6.3).

## 3. Results

### 3.1. Evaluation of Brain Activity Using Hb Data

Table 2 shows the changes in Hb data between the rest period (immediately before tooth clenching) and the tooth clenching period. During tooth clenching, oxy-Hb significantly increased in Ch9 (MFG) and Ch19 (IFG) (Table 2). Deoxy-Hb significantly decreased at Ch7 (SFG) (Table 3). In the Brodmann area (BA), Ch9 corresponds to BA46 (DLPFC). Ch7 and Ch19 correspond to BA10 (frontal pole).

### 3.2. Identification Rate of Neural Network

Table 4, Table 5 and Table 6 show the results for each group for the oxy, deoxy, and OD images, respectively. The average accuracy of identification in the evaluation using oxy images was as follows: accuracy, 86.8 ± 7.4%; recall, 86.7 ± 10.8%; specificity, 87.0 ± 9.0%; precision, 87.3 ± 8.3%; and F-value, 0.867 ± 0.078 (Table 4). The average accuracy of identification in the evaluation using deoxy images was as follows: accuracy, 76.1 ± 15.1%; recall, 76.8 ± 12.5%; specificity, 75.5 ± 19.7%; precision, 77.2 ± 17.0%; and F-value, 0.767 ± 0.137 (Table 5). The average accuracy of identification in the evaluation using OD images was as follows: accuracy, 90.3 ± 6.5%; recall, 88.1 ± 10.8%; specificity, 92.4 ± 7.8%; precision, 92.5 ± 7.1%; and F-value, 0.899 ± 0.071 (Table 6).

### 3.3. Verification of Identification Accuracy by Receiver Operating Characteristic (ROC) Curve Analysis

**Figure 6:**
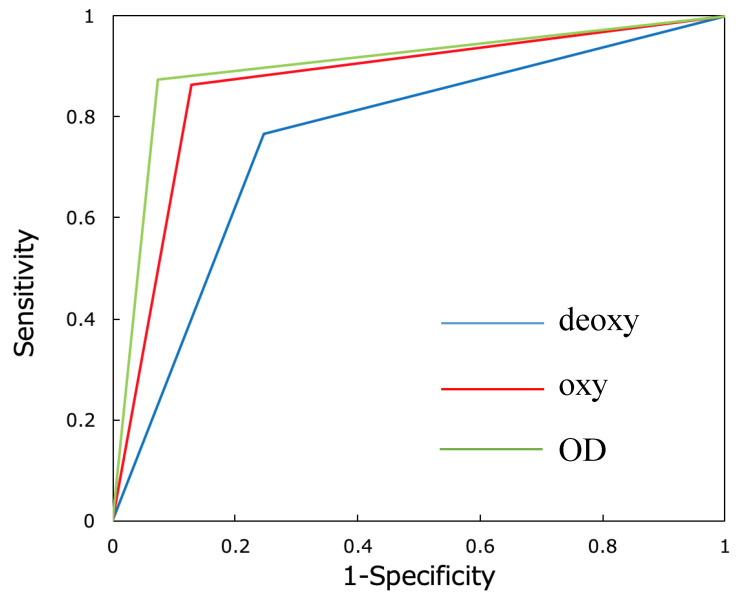
Receiver operating characteristic (ROC) curve comparison of the accuracy of the neural networks developed using oxy, deoxy, and OD images. The area under the ROC curve (AUC) related to deoxy images was 0.759 (95% confidence interval (CI): 0.750‒0.769, *p <* 0.0001), that related to oxy images was 0.867 (95% CI: 0.861‒0.874, *p* < 0.0001), and that related to OD images was 0.900 (95% CI: 0.893‒0.907, *p* < 0.0001). Deoxy: ROC curve for deep learning (DL) using deoxy images (blue line). Oxy: ROC curve for DL using oxy images (red line). OD: ROC curve for DL using OD images (green line).

The AUC (Figure 6) for the identification rate of the neural network based on deoxy images was 0.759 (95% confidence interval (CI): 0.750‒0.769, *p* < 0.0001). The AUC for the identification rate of the neural network based on oxy images was 0.867 (95% CI: 0.861‒0.874, *p* < 0.0001), while the AUC for the identification rate of the neural network based on OD images was 0.900 (95% CI: 0.893‒0.907, *p* < 0.0001).

### 3.4. Comparison of Identification Accuracy with the Number of Images Identified for Verification

The accuracy of identification of oxy images, deoxy images, and OD images by the neural network were compared. The network developed using OD images correctly identified 8502 of 9442 test images, while that developed using oxy images correctly identified 11,170 of 12,878 test images. The accuracy of the network developed using OD images was significantly higher than that of the network developed using oxy images *(p* < 0.001). The network developed using deoxy images correctly identified 8052 of 10,602 test images. The accuracy of identification was significantly higher in the network developed using OD images than in the network developed using deoxy images *(p* < 0.001). Thus, the network developed using OD images had the highest accuracy of identification.

## 4. Discussion

### 4.1. Evaluation of Brain Activity Using Hb Data

To assess the presence or absence of brain activity by fNIRS, this study used the conventional method (comparison of oxy-Hb and deoxy-Hb data at rest and during tooth clenching for each channel, using a paired *t*-test with Bonferroni post-hoc adjustment). Changes in regional cerebral blood flow and oxygen metabolism have been reported to occur due to neurovascular coupling. During neural activity, regional cerebral blood flow increases by approximately 50%, and the total oxygen consumption rate increases by approximately 5% only. Therefore, oxy-Hb increases and deoxy-Hb is washed out and reduced in activated brain areas [35]. However, other patterns of change are also observed, and in some cases, the typical patterns of increased oxy-Hb and decreased deoxy-Hb due to activation of various networks during brain activity are not shown [36,37]. In animal experiments, changes in oxy-Hb correlate best with changes in regional cerebral blood flow, so NIRS studies often use changes in oxy-Hb as indicators of neural activity [38]. In previous NIRS studies, only oxy-Hb was often mentioned [27,28,29,39]. For these reasons, we consider the data obtained in this study to constitute a dataset worth considering. In this study, oxy-Hb increased in Ch9 and Ch19 during tooth clenching, whereas deoxy-Hb decreased in Ch7. It has been reported that the lower part of the prefrontal cortex, which corresponds to the orbital region of the superior/middle/inferior frontal gyrus, is closely related to emotion and motivation, and that the DLPFC, located in the MFG, controls memory, attention, learning, and behavior monitoring. Therefore, in this study, the brain areas closely related to higher brain function were activated by tooth clenching.

### 4.2. Evaluation of Brain Activity Using DL

The data from fNIRS are a list of numerical values, which make it difficult to evaluate brain activity using only the acquired Hb data. We hypothesized that an easy visual assessment of an increase or decrease in Hb levels in brain regions, by visual presentation of the obtained data, would facilitate explanations to subjects. In addition, we speculated that the development of a program that identifies and visually presents data, and evaluates it using a network developed by DL, may make fNIRS more user-friendly.

Some psychiatric studies have examined the evaluation of Hb data using fNIRS. For example, one study proposed the use of the weighted center frequency of average waveforms of oxy-Hb data in the frontal cortex as an adjunct for the differential diagnosis of depression. The method was reported to correctly classify 74.6% of depression and 85.5% of bipolar disorder and schizophrenia cases [8]. In comparison, the accuracy of identification in the evaluation using DL on oxy images was 90% in the present study. Thus, the application of DL to fNIRS data may be promising.

In the observation of the AUCs, the use of oxy, deoxy, and OD images led to significantly accurate image identification. Networks using all three types of images also showed high identification accuracy. In particular, the comparison of the AUCs suggested that the network based on OD images yielded the highest accuracy of identification. The Ryan method, in which the numbers of correct and incorrect answers were compared, also showed that the method using OD images yielded the highest identification accuracy. Most previous studies of fNIRS analyzed oxy-Hb data only [27,28,29,39] because among Hb data obtained by fNIRS, oxy-Hb data were the most sensitive and accurate [38]. Many recent studies have simultaneously examined the behavior of oxy-Hb and deoxy-Hb data [40,41]. In this study, DL on OD data (a mixture of oxy-Hb and deoxy-Hb data) showed a higher accurate identification rate. Similar to other recent studies, the present study suggested that analyses using both oxy-Hb and deoxy-Hb data can better capture the state of brain activity than analyses using only oxy-Hb data.

On the other hand, the identification rate in one of the five groups was low. When we examined the identification rate in each subject in the group to find the cause, we found certain subjects with a significantly lower identification rate. To understand the characteristics of these subjects, we compared the changes in Hb data with those of subjects demonstrating a higher identification rate. We did not find any changes in oxy-Hb in subjects with a lower discrimination rate.

By repeating this research using DL in the future, if a stronger neural network is constructed, it will be possible to evaluate the individual subject in real time by inputting the data of the subject to be measured. We believe that this would make it possible to quickly determine the displacement of the device when it is attached and the artifacts caused by the subject′s body movement for each measurement, and to thus perform detailed analysis. Furthermore, this system can be used for rehabilitation research and clinical applications, and it is expected that explanations to patients could be easily visually explained.

### 4.3. Limitations of the Study

Most previous studies using fNIRS [27,28,29,39] have analyzed brain activity in approximately 20 subjects, a sample size that is comparable to that used in this preliminary study to develop the DL network. We obtained a relatively high identification rate of 90%, demonstrating the feasibility of DL-assisted diagnosis of brain activity during tooth clenching. However, the network developed in this study could only differentiate between rest and tooth clenching. Future studies with a larger sample size and more parameters should further improve the differentiation rate and help develop a network that identifies active channels or brain regions.

## 5. Conclusions

This preliminary study examined whether deep learning (DL) can be applied to the analysis of brain activity based on fNIRS data and showed that the network’s accurate identification rate, based on OD image data (oxy-Hb data and deoxy-Hb data), was relatively high (approximately 90%). Therefore, DL may be a useful tool for the assessment of fNIRS data.

## Figures and Tables

**Table 1:** Correspondence table for calculating the identification rate.

	Prediction Rest	Prediction Clench
Correct Rest	True-Negative	False-Positive
Correct Clench	False-Negative	True-Positive

Prediction Rest: The group identified as “at rest” by the neural network. Prediction Clench: The group identified as “tooth clenching” by the neural network. Correct Rest: Image groups for rest-period data Correct Clench: Image group for tooth clenching data True-Negative (TN): The number of images that the neural network identified as “at rest” using rest-period data. True-Positive (TP): The number of images that the neural network identified as “tooth clenching” using tooth clenching data. False-Negative (FN): The number of images that the neural network identified as “at rest” using tooth clenching data. False-Positive (FP): The number of images that the neural network identified as “tooth clenching” using rest-period data.

**Table 2:** Changes in oxy-Hb data.

Channels	Oxy-Hb Change	*p*-Value	Channels	Oxy-Hb Change	*p*-Value
Ch1	0.038 ± 0.134	6.83	Ch12	−0.043 ± 0.360	14.46
Ch2	−0.021 ± 0.151	13.27	Ch13	0.092 ± 0.252	4.22
Ch3	−0.006 ± 0.125	19.14	Ch14	0.084 ± 0.553	12.69
Ch4	0.027 ± 0.104	7.54	Ch15	0.080 ± 0.182	2.68
Ch5	0.057 ± 0.200	6.67	Ch16	−0.040 ± 0.306	12.20
Ch6	−0.006 ± 0.124	18.71	Ch17	−0.098 ± 0.540	11.19
Ch7	−0.044 ± 0.229	10.67	Ch18	0.179 ± 0.196	0.09
Ch8	−0.033 ± 0.242	13.58	Ch19	0.305 ± 0.247	0.01
Ch9	0.097 ± 0.095	0.04	Ch20	0.093 ± 0.356	7.63
Ch10	0.128 ± 0.158	0.20	Ch21	0.061 ± 0.293	9.88
Ch11	−0.032 ± 0.221	13.00	Ch22	0.132 ± 0.320	3.20

Oxy-Hb levels significantly increased in Ch9 and Ch19. Ch, channel; oxy-Hb change, mean changes, and standard deviation of oxy-Hb data averaged over the 1 min rest and tooth clenching periods; *p*-value, *p*-values for differences between rest and tooth clenching periods for each channel, calculated using paired *t*-tests, followed by Bonferroni post-hoc tests. The units of oxy-Hb and deoxy-Hb change were m(mol/L) mm.

**Table 3:** Changes in deoxy-Hb data.

Channels	Deoxy-Hb Change	*p*-Value	Channels	Deoxy-Hb Change	*p*-Value
Ch1	−0.029 ± 0.041	0.42	Ch12	−0.023 ± 0.054	3.03
Ch2	−0.025 ± 0.053	2.09	Ch13	−0.013 ± 0.059	9.23
Ch3	−0.027 ± 0.047	1.08	Ch14	0.240 ± 0.515	2.27
Ch4	−0.042 ± 0.087	2.03	Ch15	−0.041 ± 0.079	1.52
Ch5	0.043 ± 0.190	9.13	Ch16	−0.074 ± 0.108	0.47
Ch6	−0.030 ± 0.052	1.09	Ch17	−0.119 ± 0.288	3.19
Ch7	−0.045 ± 0.039	0.02	Ch18	0.078 ± 0.180	2.78
Ch8	−0.036 ± 0.048	0.30	Ch19	0.068 ± 0.126	1.40
Ch9	−0.021 ± 0.112	10.95	Ch20	−0.037 ± 0.123	6.40
Ch10	0.016 ± 0.108	12.79	Ch21	−0.028 ± 0.093	6.34
Ch11	−0.028 ± 0.051	1.32	Ch22	−0.068 ± 0.240	6.78

Deoxy-Hb levels were significantly decreased in Ch7. Ch, channel; deoxy-Hb change, mean changes, and standard deviation of deoxy-Hb data averaged over the 1 min rest and tooth clenching periods; *p*-value, *p*-values for differences between rest and tooth clenching periods for each channel, calculated using paired *t*-tests, followed by Bonferroni post-hoc tests. The units of oxy-Hb and deoxy-Hb change were m(mol/L) mm.

**Table 4:** Identification rate of the neural network based on oxy image data.

Identification RateGroups	Accuracy (%)	Recall (%)	Specificity (%)	Precision (%)	F-Value
Group A	78.0	75.1	80.9	79.7	0.773
Group B	84.4	86.0	82.9	83.4	0.847
Group C	88.1	97.9	78.4	81.9	0.892
Group D	98.4	97.3	99.5	99.5	0.984
Group E	85.3	77.2	93.5	92.2	0.840
Average ± SD	86.8 ± 7.4	86. 7 ± 10.8	87.0 ± 9.0	87.3 ± 8.3	0.867 ± 0.078

The identification rate of the neural network developed using oxy images based on the correspondence table for calculating the identification rate shown in Table 1. Accuracy = (TN + TP)/(TN + TP + FN + FP) Recall = TP/(TP + FN), Specificity = TN/(FP + TN). Precision = TP/(FP + TP) F-value = 2 × Recall × Precision/(Recall + Precision).

**Table 5:** Identification rate of the neural network based on deoxy image data.

Identification Rate Groups	Accuracy (%)	Recall (%)	Specificity (%)	Precision (%)	F-Value
Group A	56.2	62.8	49.7	55.5	0.590
Group B	86.2	90.8	81.6	83.2	0.868
Group C	90.2	88.7	91.6	91.3	0.900
Group D	84.1	73.7	94.5	93.0	0.822
Group E	64.0	67.9	60.2	63.0	0.654
Average ± SD	76.1 ± 15.1	76.8 ± 12.5	75.5 ± 19.7	77.2 ± 17.0	0.767 ± 0.137

The identification rate of the neural network developed using deoxy images based on the correspondence table for calculating the identification rate shown in Table 1. Accuracy = (TN + TP)/(TN + TP + FN + FP) Recall = TP/(TP + FN), Specificity = TN/(FP + TN). Precision = TP/(FP + TP) F-value = 2 × Recall × Precision/(Recall + Precision).

**Table 6:** Identification rate of the neural network based on OD image data.

Identification RateGroups	Accuracy (%)	Recall (%)	Specificity (%)	Precision (%)	F-Value
Group A	83.3	70.0	96.5	95.3	0.807
Group B	85.3	87.5	83.1	83.8	0.856
Group C	94.8	92.0	97.6	97.5	0.946
Group D	98.8	97.6	100	100	0.988
Group E	89.2	93.5	85.0	86.1	0.897
Average ± SD	90.3 ± 6.5	88.1 ± 10.8	92.4 ± 7.8	92.5 ± 7.1	0.899 ± 0.071

The identification rate of the neural network developed using OD images based on the correspondence table for calculating the identification rate shown in Table 1. Accuracy = (TN + TP)/(TN + TP + FN + FP) Recall = TP/(TP + FN), Specificity = TN/(FP + TN). Precision = TP/(FP + TP) F-value = 2 × Recall × Precision/(Recall + Precision).
